# Prognostic Value of Neutrophil–Lymphocyte Ratio, Platelet–Lymphocyte Ratio, and Combined Neutrophil–Lymphocyte Ratio and Platelet–Lymphocyte Ratio in Stage IV Advanced Gastric Cancer

**DOI:** 10.3389/fonc.2020.00841

**Published:** 2020-06-19

**Authors:** Huan Wang, Yongfeng Ding, Ning Li, Luntao Wu, Yuan Gao, Cheng Xiao, Haiping Jiang, Yulong Zheng, Chenyu Mao, Jing Deng, Haiyong Wang, Nong Xu

**Affiliations:** ^1^Department of Medical Oncology, First Affiliated Hospital of Zhejiang University, Hangzhou, China; ^2^Department of Surgical Oncology, The First Affiliated Hospital of Zhejiang University, Hangzhou, China

**Keywords:** neutrophil–lymphocyte ratio, platelet–lymphocyte ratio, gastric cancer, prognosis, stage IV

## Abstract

**Background:** The prognostic value of neutrophil–lymphocyte ratio (NLR), platelet–lymphocyte ratio, and the combined NLR–PLR score in patients with stage IV gastric carcinoma (GC) has not yet been clarified. Therefore, this study aimed to explore the potential association of NLR, PLR, and NLR–PLR score with the prognosis of patients with stage IV GC.

**Methods:** This retrospective study included 466 patients with GC diagnosed between 2010 and 2017. High NLR and high PLR were defined using the median values as the cutoff values. We then combined the NLR and PLR value and generated the NLR–PLR score as a new biomarker. Patients were divided into three groups according to their NLR–PLR score. Univariate and multivariate analyses were conducted to compare survival outcomes.

**Results:** Median overall survival (OS) and progression-free survival (PFS) were 15.5 months (range, 0.7–96.8 months) and 6.7 months (range, 0.5–30.4 months), respectively. The NLR, PLR, and the NLR–PLR scores were correlated with clinical outcomes such as OS and PFS. Median OS for patients with NLR–PLR scores of 0, 1, and 2 was 22.5, 15.7, and 11.2 months, respectively. Median PFS for patients with these NLR–PLR scores of 0, 1, and 2 was 7.8, 7.1, and 5.2 months, respectively (*P* < 0.001). High NLR–PLR scores predicted poor survival in patients with stage IV GC (all *P* < 0.05).

**Conclusion:** Our findings provide scientific evidence to support that the NLR–PLR score may be able to independently predict survival outcomes in patients with stage IV GC.

## Introduction

Gastric carcinoma (GC) is one of the most common malignant tumors of the digestive system (1). Because GC is highly heterogeneous and malignant, early diagnosis and prediction of treatment outcomes, recurrence, and metastasis are challenging. Several patients are diagnosed during a later stage of disease or develop recurrence after surgery. Therefore, prognostic biomarkers are needed to stratify patients who may benefit from treatment.

Studies have reported that inflammation is the primary cause of tumorigenesis (2, 3). Inflammatory cytokines such as IL-6 are involved in tumor progression and metastasis (4, 5), and experimental studies reported that inflammation can initiate cancer (3, 6). Neutrophils comprise the majority of leukocyte components in the peripheral blood circulation and have an important impact on immunity. Furthermore, inflammatory mediators produced by neutrophils may modulate the tissue and tumor microenvironment (TME) and promote tumor development, angiogenesis, progression, and metastasis (7, 8). However, lymphocytes could cause cytotoxic cell death, produce inhibitive cytokines, and regulate tumor cell action. Therefore, fewer lymphocytes may lead to fewer immunological responses to malignancies, ultimately resulting in poorly controlled suppression of tumor proliferation (9). Platelets have an impact on tumor proliferation and metastasis and significant roles in cancers; however, the potential mechanisms remain unclear (10). Notably, a recent study demonstrated that platelet-derived signals were necessary for the recruitment of granulocytes, which could further contribute to the formation of early metastatic niches for tumor cells (11).

Neutrophils, platelets, and lymphocytes have crucial roles in tumor-related inflammation and immunology; therefore, their levels have prognostic value (12, 13). Several studies have demonstrated that the neutrophil–lymphocyte ratio (NLR; defined as the neutrophil count divided by the lymphocyte count) and the platelet–lymphocyte ratio (PLR; defined by dividing the number of platelets by the number of lymphocytes) have significant value regarding prognosis, especially digestive system and gynecologic and lung cancers (14–20). However, the prognostic value of NLR and PLR for advanced GC is unclear. This study aimed to analyze the clinical value of NLR, PLR, and the combined NLR–PLR score as novel predictors of advanced GC.

## Materials and Methods

### Patients

This retrospective study included stage IV GC patients diagnosed at the Medical Oncology Department of the First Affiliated Hospital of Zhejiang University, Republic of China between 2010 and 2017. The inclusion criteria were pathologically and clinically confirmed stage IV GC and available results for routine blood tests before first-line treatment. The exclusion criteria were as follows: hepatitis B; immune system diseases including rheumatic immune system disease, systemic lupus erythematosus, rheumatoid arthritis, Sjogren's syndrome, Behcet's disease, systemic vasculitis, gout, dermatomyositis, arthritis, ankylosing spondylitis, acquired immune deficiency syndrome, and syphilis; infectious diseases (determined according to the use of antibiotics not indicated for invasive surgical prophylaxis documented in the electronic medical system); first-line chemotherapy outside the study setting; pre-treatment blood count values not obtained within a week before the initiation of first-line chemotherapy; incomplete medical record information; and missing follow-up information.

The collection and analysis of all samples in this study were approved by the Ethics Committee of the First Affiliated Hospital of Zhejiang University (reference number: 2017–802).

### Data Collection

Clinicopathological findings and follow-up status were documented. Neutrophil, lymphocyte, and platelet values before first-line chemotherapy were recorded. Fasting venous blood (total, 2 ml blood) was collected in an the EDTA-K2 anticoagulant tube in the morning. All blood specimens were analyzed using the Sysmex XN-1000 blood analyzer. The first-line chemotherapy regimen was defined as follows: the first-line chemotherapy regimen administered after the initial diagnosis of stage IV advanced GC; the first-line chemotherapy regimen administered since recurrence that occurred more than 6 months after the completion of postoperative adjuvant chemotherapy; the adjuvant chemotherapy regimen administered after recurrence within 6 months was considered as the first-line chemotherapy regimen. Staging was performed according to the Eighth American Joint Committee on Cancer TNM staging system. Intra-abdominal metastasis was defined as visceral organ or peritoneal metastasis. All laboratory values were measured within 1 week before the first-line chemotherapy. All data were collected from the electronic medical records system.

### Evaluation of the NLR, PLR, and NLR–PLR Scores

The pre-treatment laboratory peripheral blood examinations of platelet counts, lymphocyte counts, and neutrophil counts were recorded to calculate NLR and PLR scores. NLR was calculated as the neutrophil count divided by the lymphocyte count. PLR was calculated by dividing the number of platelets by the number of lymphocytes. NLR and PLR were then categorized as high or low using the median values as the cutoff (2.8 for NLR and 174.79 for PLR). To perform better stratification of patients with different risks, we classified them into three groups according to their NLR–PLR scores: low NLR and low PLR indicated an NLR–PLR score of 0; high NLR and high PLR indicated an NLR–PLR score of 2; high NLR or high PLR indicated an NLR–PLR score of 1.

### Follow-Up

The primary endpoints were overall survival (OS) and progression-free survival (PFS). OS was defined as the interval between the date of first-line chemotherapy and death from any cause. PFS was defined as the time from the date of the initial first-line chemotherapy to the time of the first recurrence or metastasis after first-line chemotherapy. Patients were followed up every 3 months until death.

### Statistical Analysis

All statistical analyses were performed using GraphPad Prism 6 and IBM SPSS Statistics version 20.0 software. The chi-squared test or the Fisher's exact test was performed to compare the relationships among NLR, PLR, and other variables. The survival curve was plotted using the Kaplan–Meier method. The log-rank test was used to analyze the differences between the survival curves. Univariate and multivariate analyses of prognostic factors were conducted using Cox's proportional hazards model. *P*-values < 0.05 were considered to indicate statistical significance.

## Results

### Patient Characteristics

**Figure 1:**
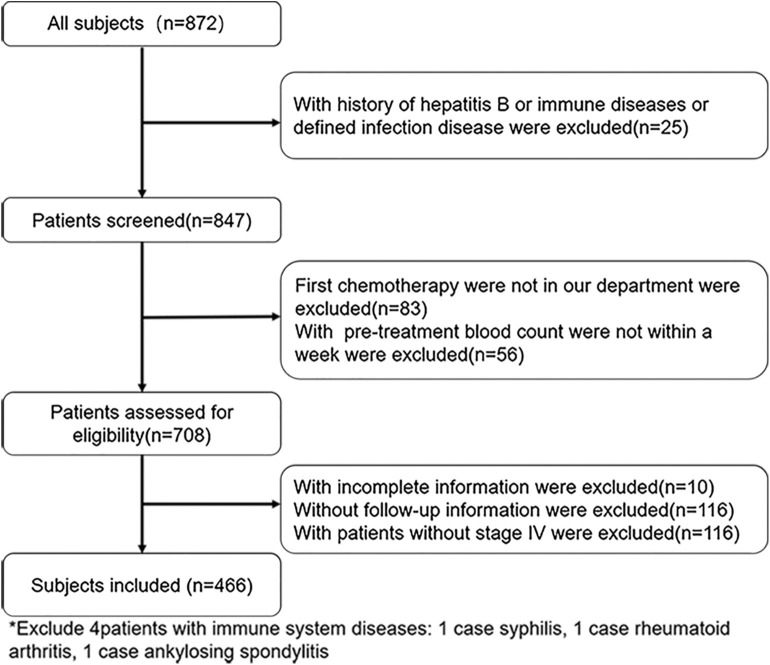
Flowchart presenting the steps of inclusion and exclusion of subjects.

Of the 872 patients initially identified, we excluded 406 due to hepatitis B infection (*n* = 10), immune system diseases (*n* = 3), infection (*n* = 12), first-line chemotherapy outside our department (*n* = 83), pre-treatment blood count values not measured within 1 week before the first-line chemotherapy (*n* = 56), incomplete medical record information (*n* = 10), missing follow-up information (*n* = 116), and non-stage IV malignancy (*n* = 116). Finally, 466 patients were included for further analysis (Figure 1). Table 1 summarizes the patient characteristics. Patients were divided into NLR <2.8 group (*n* = 235), NLR ≥2.8 group (*n* = 231), PLR <174.79 group (*n* = 233), and PLR ≥174.49 group (*n* = 233). The cohort comprised 327 (70.2%) men and 139 (29.8%) women, with a median age of 60 years (range, 20–88 years). A total of 200 (42.9%) patients had a history of surgery for GC and 219 (47.0%) had intra-abdominal metastasis. A total of 190 (40.8%) patients had high carcinoembryonic antigen (CEA) levels (upper limit of the normal range according to this hospital, ≥5 U/ml) and 181 (38.8%) patients had high Carbohydrate Antigen 199 (CA199) levels (upper limit of normal range according to this hospital, ≥37 U/ml). There were 228 (48.9%) patients with poor differentiation. The median OS and PFS were 15.5 months (range, 0.7–96.8 months) and 6.7 months (range, 0.5–30.4 months), respectively.

**Table 1:** Clinical characteristics of the patients with different NLR and PLR.

**Group**	**No. of patients (*N =* 466)**	**NLR < 2.8 (*n =* 235)**	**NLR≥2.8 (*n =* 231)**	***P*-value**	**PLR < 174.79 (*n =* 233)**	**PLR≥174.79 (*n =* 233)**	***P*-value**
Age							
<60	243 (52.1%)	126 (53.6%)	117 (50.6%)	0.521	113 (48.5%)	130 (55.8%)	0.115
≥.1	223 (47.9%)	109 (46.4%)	114 (49.4%)		120 (51.5%)	103 (44.2%)	
Sex							
Female	139 (29.8%)	70 (29.8%)	69 (29.9%)	0.984	52 (22.3%)	87 (37.3%)	<0.001
Male	327 (70.2%)	165 (70.2%)	162 (70.1%)		181 (77.7%)	146 (62.7%)	
History of gastric cancer operation							
Yes	200 (42.9%)	139 (59.1%)	61 (26.4%)	<0.001	113 (48.5%)	87 (37.3%)	0.015
No	266 (57.1%)	96 (40.9%)	170 (73.6%)		120 (51.5%)	146 (62.7%)	
Intra-abdominal metastasis							
Yes	219 (47.0%)	92 (39.1%)	127 (55.0%)	0.001	97 (41.6%)	122 (52.4%)	0.020
No	247 (53.0%)	143 (60.9%)	104 (45.0%)		136 (58.4%)	111 (47.6%)	
History of smoke							
Yes	167 (35.8%)	87 (37.0%)	80 (34.6%)	0.591	99 (42.5%)	68 (29.2%)	0.003
No	299 (64.2%)	148 (63.0%)	151 (65.4%)		134 (57.5%)	165 (70.8%)	
History of alcohol							
Yes	139 (29.8%)	75 (31.9%)	64 (27.7%)	0.321	77 (33.0%)	62 (26.6%)	0.129
No	327 (70.2%)	160 (68.1%)	167 (72.3%)		156 (67.0%)	171 (73.4%)	
Hypertension							
Yes	78 (16.7%)	34 (14.5%)	44 (19.0%)	0.185	40 (17.2%)	38 (16.3%)	0.804
No	388 (83.3%)	201 (84.5%)	187 (81.0%)		193 (82.8%)	195 (83.7%)	
Diabetes							
Yes	31 (6.7%)	16 (6.8%)	15 (6.5%)	0.891	15 (6.4%)	16 (6.9%)	0.853
No	435 (93.3%)	219 (93.2%)	216 (93.5%)		218 (93.6%)	217 (93.1%)	
CEA							
<5	276 (59.2%)	154 (65.5%)	122 (52.8%)	0.005	142 (60.9%)	134 (57.5%)	0.451
≥5	190 (40.8%)	81 (34.5%)	109 (47.2%)		91 (39.1%)	99 (42.5%)	
CA199							
<37	285 (61.2%)	160 (68.1%)	125 (54.1%)	0.002	153 (65.7%)	132 (56.7%)	0.046
≥37	181 (38.8%)	75 (31.9%)	106 (45.9%)		80 (34.3%)	101 (43.3%)	
BMI							
<18.5	76 (16.3%)	40 (17.0%)	36 (15.6%)	0.859	27 (11.6%)	49 (21.0%)	0.021
18.5–24.9	356 (76.4%)	177 (75.3%)	179 (77.5%)		187 (80.3%)	169 (72.5%)	
≥25	34 (7.3%)	18 (7.7%)	16 (6.9%)		19 (8.2%)	15 (6.4%)	
Differentiation							
Poor	228 (48.9%)	117 (49.8%)	111 (48.1%)	0.708	112 (48.1%)	116 (49.8%)	0.711
Moderate-Well	238 (51.1%)	118 (50.2%)	120 (51.9%)		121 (51.9%)	117 (50.2%)	

### Association Between NLR or PLR Levels and Clinicopathological Variables

High NLR was significantly associated with no history of GC surgery (*P* < 0.001), intra-abdominal metastasis (*P* = 0.001), high CEA levels (*P* = 0.005), and high CA199 levels (*P* = 0.002). Meanwhile, high PLR was significantly associated with female sex (*P* < 0.001), no history of GC surgery (*P* = 0.015), intra-abdominal metastasis (*P* = 0.020), no history of smoking (*P* = 0.003), high CA199 levels (*P* = 0.046), and body mass index (BMI) < 18.5 kg/m^2^ (*P* = 0.021).

### Prognostic Significance of NLR or PLR

**Figure 2:**
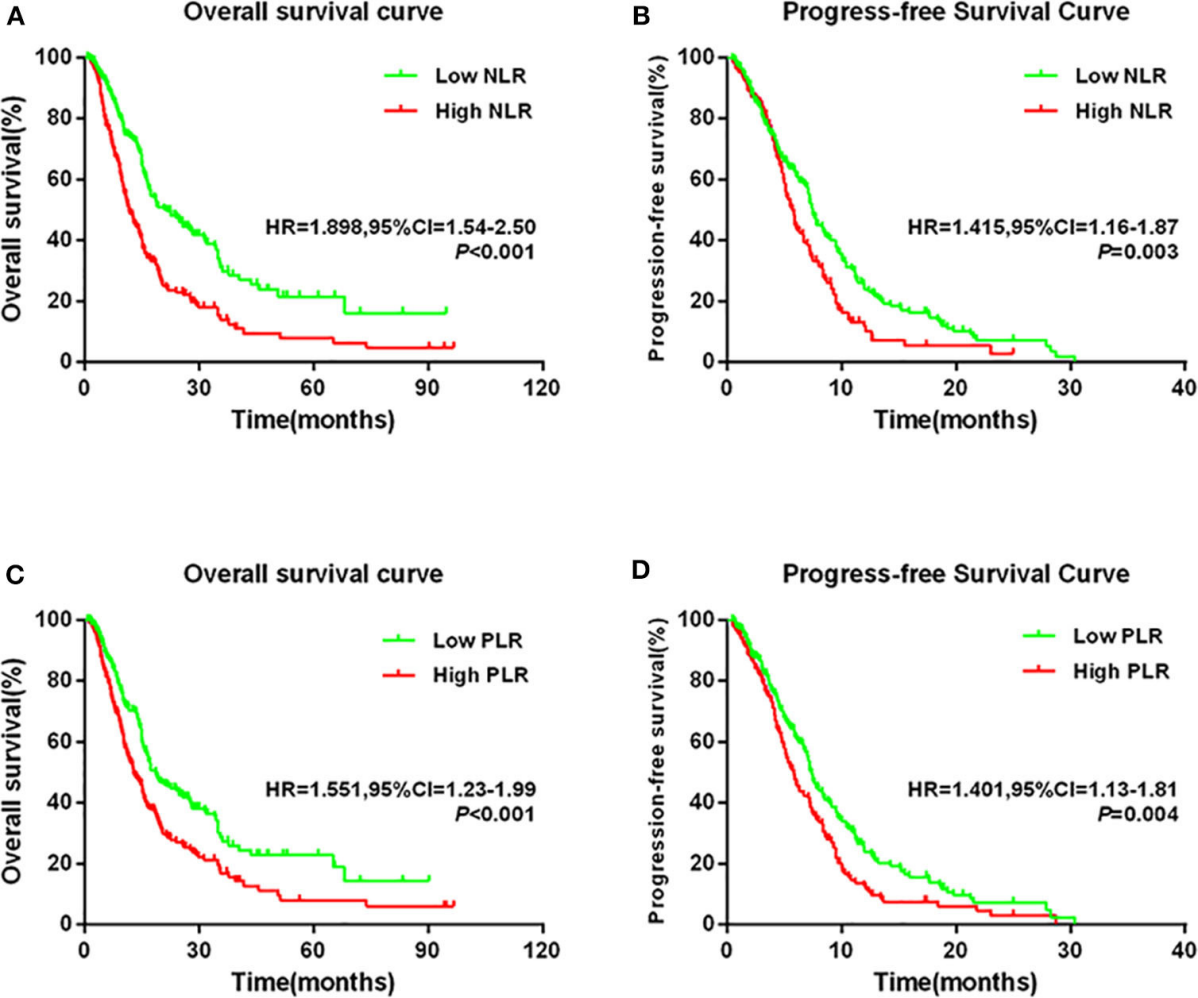
Kaplan–Meier survival curves in patients stratified by NLR or PLR median for **(A)** OS for NLR median, **(B)** PFS for NLR median, **(C)** OS for PLR median, and **(D)** PFS for PLR median.

Kaplan–Meier analysis and log-rank test demonstrated that compared with low levels, high NLR levels or PLR levels significantly predicted poorer OS and PFS (Figure 2). Compared with the low NLR group, the high NLR group had significantly shorter 5-year OS rate (7.9% vs. 21.5%) and median OS time (11.6 months vs. 21.5 months) [hazard ratio (HR) = 1.898, 95% confidence interval (CI) = 1.54–2.50; *P* < 0.001; Figure 2A]. Moreover, the high-NLR group had significantly shorter median PFS time than the low-NLR group (5.7 vs. 7.5 months; HR = 1.415, 95% CI = 1.16–1.87; *P* = 0.003; Figure 2B). With respect to PLR, the high-PLR group had significantly shorter 5-year OS rate and median OS time than did the low-PLR group (7.9% vs. 22.8%; 12.8 vs. 18.6 months; HR = 1.551, 95% CI = 1.23–1.99; *P* < 0.001; Figure 2C). Meanwhile, the high-PLR group had significantly shorter median PFS than the low-PLR group (5.8 vs. 7.4 months; HR = 1.401, 95% CI = 1.13–1.81; *P* = 0.004; Figure 2D).

### Univariate and Multivariate Survival Analyses of Prognostic Factors

Univariate Cox proportional hazards analyses revealed that history of GC operation (yes vs. no, *P* < 0.001), CEA level (<5 vs. ≥5 U/ml, *P* = 0.071), CA199 level (<37 vs. ≥37 U/ml, *P* = 0.001), and NLR (<2.8 vs. ≥2.8, *P* < 0.001) were significantly associated with OS (Supplementary Table 1). Therefore, they were included in the multivariate Cox proportional hazards model, along with tumor differentiation parameters. Multivariate analyses revealed that history of GC operation (HR = 0.615, 95% CI = 0.47-−0.80; *P* < 0.001), NLR level (HR = 1.674, 95% CI = 1.30–2.16; *P* < 0.001), and tumor differentiation (HR = 0.735, 95% CI = 0.58–0.94; *P* = 0.012) were the independent prognostic indicators for patients with stage IV GC. Similarly, univariate Cox proportional hazards analyses also indicated that PLR was significantly associated with OS (HR = 1.555, 95% CI = 1.23–1.97; *P* < 0.001) (Supplementary Table 2). Multivariate Cox proportional hazards analyses revealed that history of GC (HR = 0.537, 95% CI = 0.42–0.69; *P* < 0.001), PLR (HR = 1.483, 95% CI = 1.17–1.89; *P* = 0.001), and tumor differentiation (HR = 0.741, 95% CI = 0.58–0.94; *P* = 0.015) were the independent prognostic indicators of stage IV GC.

### Subgroup Analyses of the Prognostic Value of NLR or PLR Alone

Patients were divided into two groups according to their median value (younger than 60 years and 60 years or older). Based on the normal range set by the First Affiliated Hospital of Zhejiang University, CEA or CA199 levels were divided into two groups (high and low). Differentiation was divided into two groups (poor and moderated-well) on the basis of the pathological report. BMI was divided into three groups (<18.5 kg/m^2^, 18.5–24.9 kg/m^2^, and ≥25 kg/m^2^) according to the recommendation of the World Health Organization (WHO).

**Figure 3:**
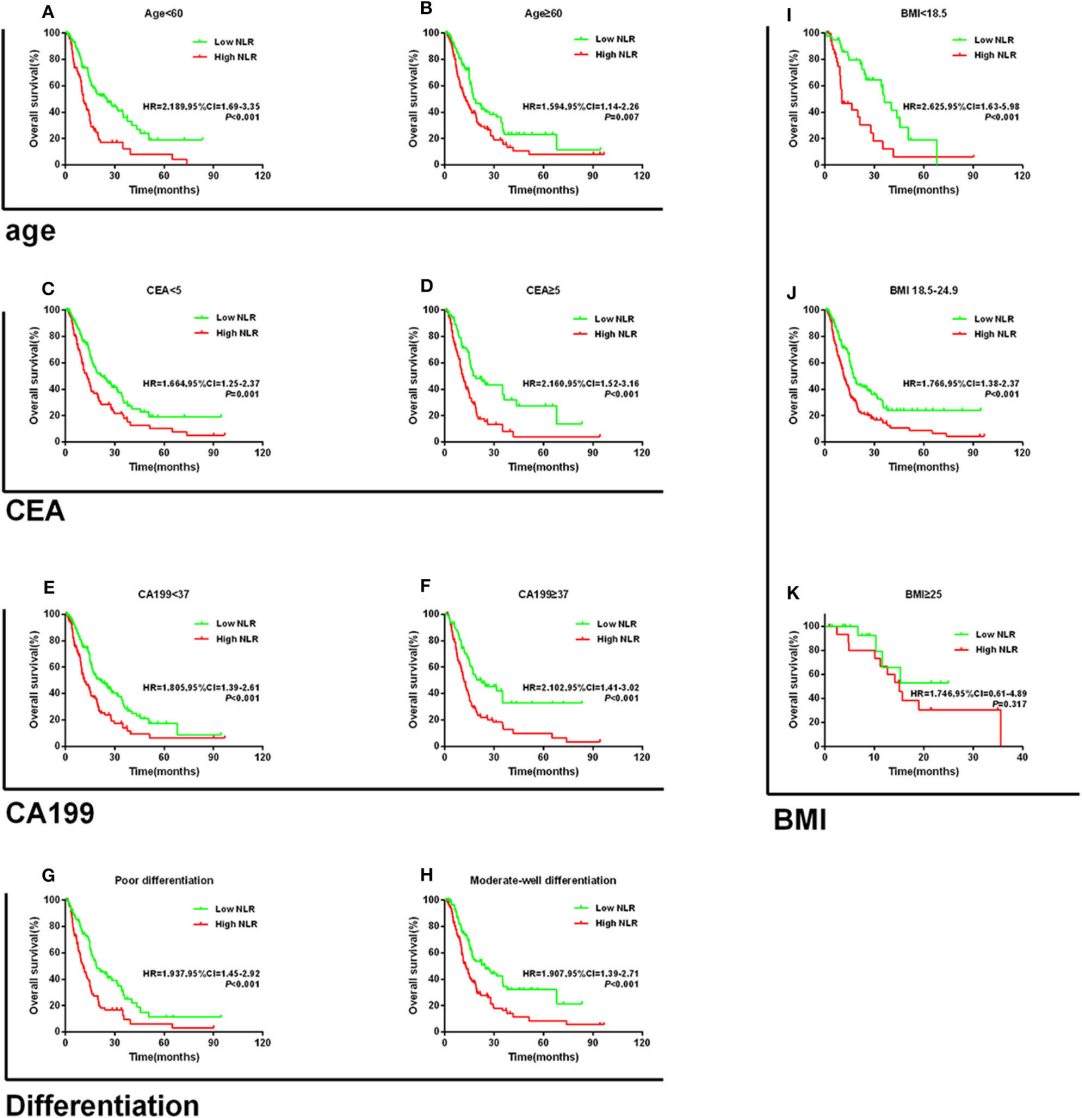
Kaplan–Meier survival curves for overall survival in patients stratified by NLR median for **(A)** age < 60 and **(B)** age ≥ 60; **(C)** CEA < 5; **(D)** CEA ≥ 5; **(E)** CA199 < 37; **(F)** CA199 ≥ 37; **(G)** poor differentiation; **(H)** moderate-well differentiation; **(I)** BMI < 18.5; **(J)** BMI 18.5–24.9; and **(K)** BMI ≥ 25.

When stratified by age, the prognostic value of NLR was still maintained for those aged <60 years (HR = 2.189, 95% CI = 1.69–3.35; *P* < 0.001; Figure 3A) and ≥60 years (HR = 1.594, 95% CI = 1.14–2.26; *P* = 0.007; Figure 3B). Similarly, high NLR was still a worse prognostic indicator in the CEA, CA199, and differentiation subgroups (all *P* < 0.05, Figures 3C–H). In addition, we found that high NLR predicted poor OS in both the BMI <18.5 kg/m^2^ and the BMI 18.5–24.9 kg/m^2^ groups (all *P* < 0.05; Figures 3I,j). However, NLR had no significant prognostic effect in the BMI ≥25 group (*P* = 0.317; Figure 3K). Consistent results were obtained regarding the prognostic effects of PLR for the aforementioned subgroups (CEA, CA199, and differentiation). Similarly, when stratified by age (<60 or ≥60), CA199 (<5 or ≥5), differentiation (poor or moderated-well), and BMI (<18.5, 18.5–24.9, or ≥25), patients with high PLR scores had worse prognosis (all *P* < 0.05; Supplementary Figures 1A,B,E–K). Furthermore, we found that high PLR was significantly associated with poorer OS in the patients with CEA ≥5 U/ml (HR = 1.867, 95% CI = 1.31–2.73, *P* < 0.001; Supplementary Figure 1C); however, inconsistent results were obtained for the subgroup with CEA <5 (HR = 1.353, 95% CI = 0.99–1.86; *P* = 0.053; Supplementary Figure 1D).

### Prognostic Value of the NLR–PLR Score

**Figure 4:**
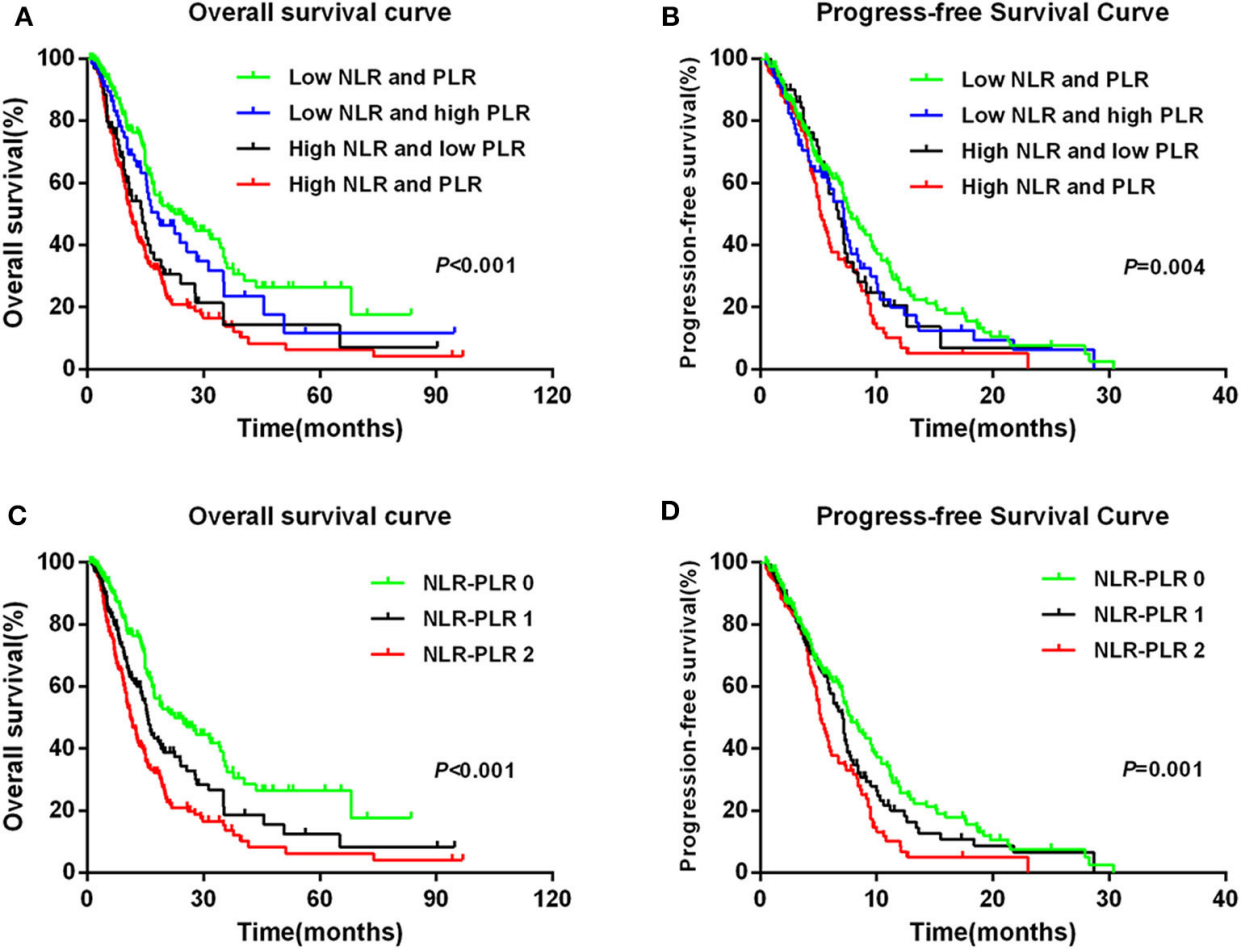
Kaplan–Meier survival curves in patients: NLR + PLR with four groups for **(A)** OS and **(B)** PFS; NLR–PLR score for **(C)** OS and **(D)** PFS.

To further explore whether patients with different NLR and PLR according to their dichotomized values had different prognoses, we classified patients into four groups according to the NLR and PLR levels as follows: the low-NLR and low-PLR group; the low-NLR and high-PLR group; the high-NLR and low-PLR group; and the high-NLR and high-PLR group. We found that the high-NLR and high-PLR group had the worst prognosis for OS and PFS and that the low-NLR and low-PLR group had the best prognosis (Figures 4A,B).

To better stratify patients, we reintegrated patients into three groups according to NLR–PLR scores. A total of 165 (35.4%) patients had an NLR–PLR score of 0; 138 (29.6%) patients had an NLR–PLR score of 1; and 163 (35.0%) patients had an NLR–PLR score of 2. The 5-year OS rates for patients with NLR–PLR scores of 0, 1, and 2 were 26.5, 12.5, and 6.2%, respectively (*P* < 0.001; Figure 4C); however, the median OS times were 22.5, 15.7, and 11.2 months, respectively. Median PFS times for patients with NLR–PLR scores of 0, 1, and 2 were 7.8, 7.1, and 5.2 months, respectively (*P* < 0.001; Figure 4D). The NLR–PLR score was identified as an independent prognostic factor for OS in the multivariate model (*P* < 0.001; Table 2). Thus, we used the three groups to analyze the prognostic value of the NLR–PLR score in the subgroup analyses.

**Table 2:** Univariate and multivariate Cox regression analysis for overall survival according to combined NLR-PLR.

	**Univariate**		**Multivariate**	***P*-value**
	**HR (95%CI)**	**P-value**	**HR (95%CI)**	
Age (<60 vs ≥60)	0.984 (0.78–1.25)	0.892		
Sex (female vs male)	1.00 (0.78–1.30)	0.973		
History of gastric cancer operation (no vs. yes)	0.543 (0.43–0.69)	<0.001	0.589 (0.46–0.76)	<0.001
Intra-abdominal metastasis (no vs yes)	1.061 (0.84–1.35)	0.624		
History of smoke (no vs. yes)	1.128 (0.88–1.44)	0.335		
History of alcohol (no vs. yes)	1.037 (0.80–1.34)	0.779		
Hypertension (no vs. yes)	1.116 (0.81–1.53)	0.496		
Diabetes (no vs. yes)	1.352 (0.86–2.11)	0.185		
CEA (<5 vs. ≥5)	1.248 (0.98–1.59)	0.071	1.163 (0.91–1.49)	0.231
CA199 (<37 vs. ≥37)	1.070 (0.84–1.37)	0.001	0.902 (0.70–1.16)	0.426
NLR-PLR	Ref	<0.001	Ref	<0.001
NLR-PLR(0/1)	1.525 (1.12–2.08)	0.007	1.487 (1.09–2.03)	0.012
NLR-PLR(0/2)	2.158 (1.62–2.87)	<0.001	1.886 (1.40–2.54)	<0.001
Differentiation (poor vs. moderate-well)	0.830 (0.66–1.05)	0.124	0.735 (0.58–0.94)	0.012

### Subgroup Analysis of the Prognostic Value of the NLR–PLR Score

The subgroup analysis of the prognostic value of the NLR–PLR score in stage IV GC was conducted according to age, CEA level, CA199 level, BMI, type of differentiation, and first-line chemotherapy regimen. When stratified by age, the NLR–PLR score still had a prognostic value for those aged <60 years (*P* < 0.001; Supplementary Figure 2A) and age ≥60 years (*P* = 0.019; Supplementary Figure 2B). Furthermore, the NLR–PLR score also effectively stratified the OS of patients irrespective of CEA level, CA199 level, BMI, and type of differentiation (all *P* < 0.05; Supplementary Figures 2C–K).

**Figure 5:**
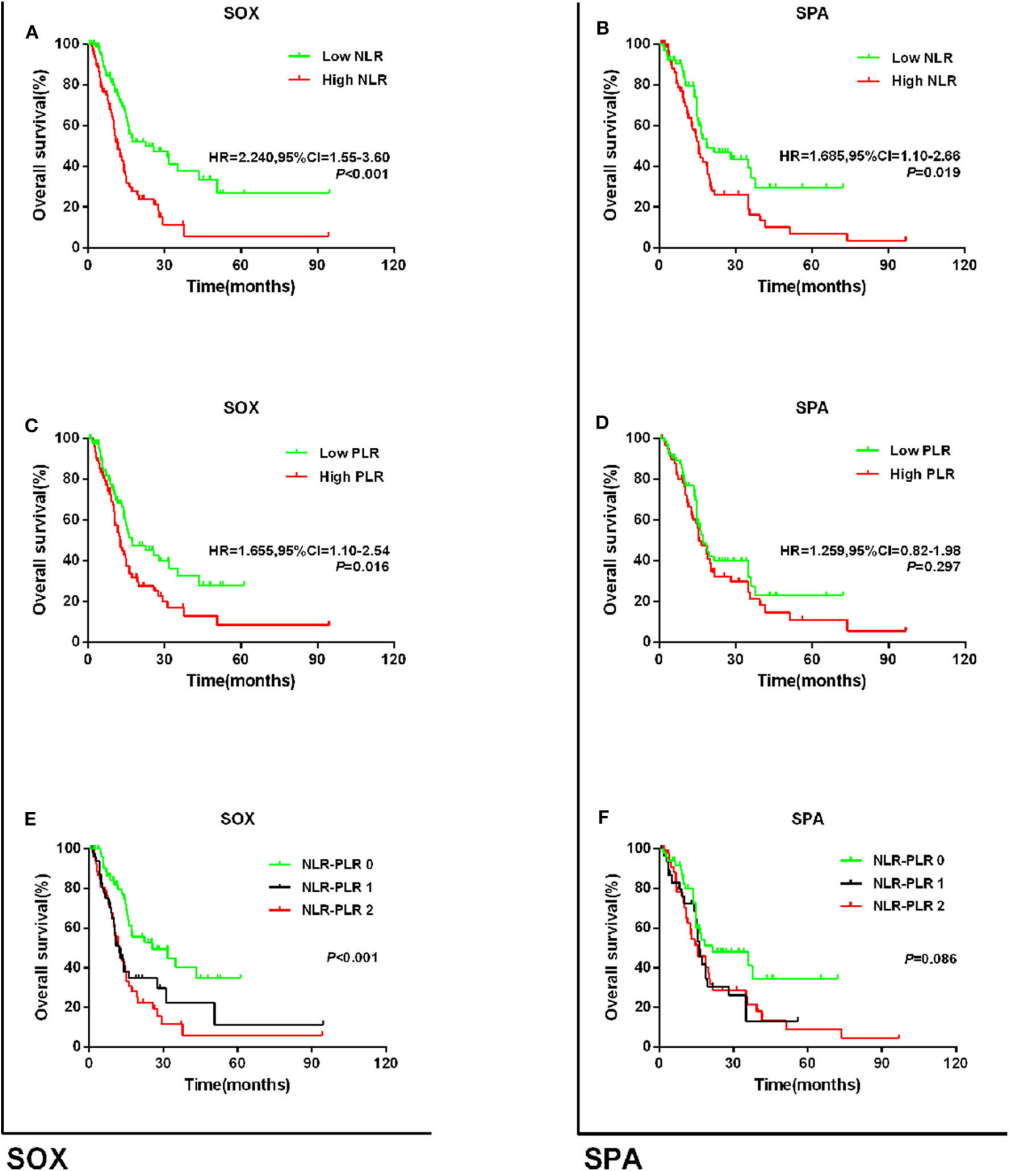
Kaplan–Meier survival curves for overall survival in patients stratified by NLR or PLR median for **(A)** the relationship of NLR and OS via SOX subgroup; **(B)** the relationship of PLR and OS via SOX subgroup; **(C)** the relationship of NLR + PLR and OS via SOX subgroup; **(D)** the relationship of NLR and OS via SPA subgroup; **(E)** the relationship of PLR and OS via SPA subgroup; and **(F)** the relationship of NLR + PLR and OS via SPA subgroup.

Regarding the first-line chemotherapy regimen, we stratified patients into those who received S-1 plus oxaliplatin (SOX) and those who received S-1 plus paclitaxel (SPA). Among the patients who received the SOX regimen, those with high NLR or high PLR had poorer OS than those with low NLR or low PLR (NLR: HR = 2.240, 95% CI = 1.55–3.60; *P* = 0.001; PLR: HR = 1.655, 95% CI = 1.10–2.54; *P* = 0.016; Figures 5A,B). The 5-year OS rates for patients with NLR–PLR scores of 0, 1, and 2 were 34.5, 11.1, and 5.8%, respectively (*P* < 0.001; Figure 5C), and the median OS times were 25.6, 12.1, and 12.5 months, respectively. Among those who received the SPA regimen, those with high NLR had worse OS than those with low NLR (HR = 1.685, 95% CI = 1.10–2.66; *P* = 0.019; Figure 5D). However, there were no significant differences in the OS of patients with low PLR and high PLR (HR = 1.259, 95% CI = 0.82–1.98; *P* = 0.297; Figure 5E). Patients with an NLR–PLR score of 0 tended to have better 5-year OS rates (scores of 0, 1, and 2: 34.4, 13.0, and 9.0%, respectively; *P* = 0.086; Figure 5F), and the median OS times were 21.5, 16.2, and 15.5 months, respectively.

## Discussion

This study investigated the prognostic value of NLR and PLR alone and the combined NLR–PLR score for patients with stage IV GC. The results revealed that low NLR or low PLR predicts longer survival time for these patients. Further, history of GC operation, intra-abdominal metastasis, CEA level ≥5 U/ml, CA199 level ≥37 U/ml, poor differentiation, and high NLR–PLR scores were independent prognostic factors of shorter OS. Meanwhile, patients who had characteristic of low NLR–PLR score were more likely to have longer 5-year OS rates. Subgroup analyses based on age, CA199 level, and type of differentiation showed that low NLR was significantly associated with longer OS among the subgroups. Furthermore, those with low PLR and low combined NLR–PLR score had longer OS. However, in the CEA subgroup, high NLR and high combined NLR–PLR score, but not high PLR alone, were significantly associated with worse OS (*P* = 0.053). In clinical practice, CEA is commonly used as a biomarker for predicting therapeutic effects on gastrointestinal tumors, and their elevated levels in serum are correlated with poor survival for cancer (21). Whether the inflammation cytokines could enhance the prognostic value of CEA for patients with GC requires more exploration.

Previous studies showed that low BMI (<18.5 kg/m^2^) was associated with postoperative complications among stage IV GC patients. Moreover, tumor growth could induce systemic inflammatory and comorbid diseases, which required excessive nutritional consumption, and it may cause some related poor manifestations such as cachexia (22, 23). NLR, PLR, and NLR–PLR score significantly influenced the survival time stratified according to BMI (<18.5 kg/m^2^ vs. ≥18.5 kg/m^2^). However, NLR was not related to OS in the high-BMI group. Unfortunately, the mechanism about the relationship between high NLR or high PLR and worse OS for patients with low BMI has not been clarified. A systematic review and meta-analysis of 100 studies and 40,559 patients with various solid malignant tumors concluded that higher NLR was related to worse OS (24). A meta-analysis of 20 studies and 12,754 patients demonstrated that higher PLR was associated with worse OS for those with various solid tumors (25). Some studies reported that high NLR was also related to poor OS (26) or poor disease-specific survival (27). A Japanese study reported that NLR was correlated with the survival period and was an independent predictor of OS for those with unresectable GC (28). Ramos-Esquivel et al. (29) concluded that NLR ≥ 5 and PLR ≥ 350 were associated with shorter disease-free survival and poor OS in Hispanic patients with GC. In contrast, some studies reported that there was no significant correlation between preoperative NLR or PLR and survival time for patients with early GC (30). The clinical value of NLR or PLR as an independent predictor of GC prognosis is still controversial. Furthermore, few studies have focused on the relationship between stage IV GC prognosis and NLR or PLR, and research regarding whether NLR or PLR can be used to stratify patients who will benefit from first-line chemotherapy is scarce.

Regarding the development of immunotherapy, it should be determined whether NLR or PLR is equally applicable to patients who undergo immunotherapy alone or immunotherapy combined with chemotherapy. Immunotherapy has been applied for several solid tumors. Immunological checkpoint inhibitors of anti-cytotoxic T lymphocyte antigen-4 (CTLA-4) and anti-programmed cell death-1 (PD-1) have already demonstrated remarkable clinical efficacy for solid tumors and longer survival times, but not all patients will benefit from it (31, 32). Studies have shown that immunological checkpoint inhibitors can affect the TME by establishing mice tumor models (33–36). Changes in the intracellular immune cell subsets were detected and observed in melanoma patients after treatment with Nivolumab, and the results revealed that there was no significant difference in the changes in neutrophils from melanoma patients who did not receive immunotherapy (34). However, it has been shown that the programmed cell death-ligand 1 (PD-L1) is correlated with the immunosuppressive phenotype (37, 38). However, there are still limitations about the effects of immunological checkpoint inhibitors and inflammatory cytokines.

To the best of our knowledge, there have been no studies of the prognostic value of NLR–PLR scores in stage IV GC. The mechanism behind the correlation between high NLR–PLR and poor survival time has not been clarified. However, some studies reported several potential mechanisms based on the association of NLR or PLR with inflammation (39, 40). NLR was significantly correlated with the inflammatory TME created by tumor-related macrophages and IL-17-producing cells (39). One study revealed that high NLR reflects lymphocytopenia, which impairs the host immune response to malignancy (40). Neutrophils release vascular endothelial growth factor through degranulation, thus leading to tumor growth (41). Dynamic changes in the values of tumor necrosis factor-alpha and IL-6 could reflect the cancer prognosis, depending on the tumor type, clinical progression, and cancer therapy (42). Lymphocytopenia leads to an immunosuppressive state, which is found in the majority of patients with advanced cancer (43). The density of CD4+ immune cells in the TME was decreased in patients with high NLR, who had a worse OS (44). This might be due to the increased susceptibility of lymphocyte T cells to apoptosis, resulting in the upregulation of death receptors, and related to the state of chronic activation (45), leading to lower immune response activity in tumor antigens released by cancer cells during chemotherapy (46). Platelets provide a procoagulant surface that facilitates amplification of cancer-related coagulation and can be recruited to cover tumor cells, thereby shielding them from immune responses and facilitating cancer growth and dissemination (47). Platelets and their precursors can promote an increased vascular endothelial growth factor load and inhibit the immune inflammation environment, such as that during an immune attack (48). A study revealed that distal tumors could remodel bone structure via circulating platelets (49). Platelets can shield circulating tumor cells (CTCs) from immune attack and destruction by activated platelets, which in turn protect the CTCs from shearing stresses during circulation (47). Thus, we predicted that an increase in the platelet count or neutrophil count and decrease in the lymphocyte count in the peripheral venous blood are associated with tumor development and metastasis in patients with stage IV GC.

Furthermore, we also investigated the relationship between NLR or PLR alone or the combined NLR–PLR score and prognosis according to the SOX regimen or SPA regimen as first-line chemotherapy. The results indicated that, with both regimens, patients with low NLR and low NLR–PLR scores had longer OS than those with high NLR and high NLR–PLR score. Among the patients who received the SOX regimen, those with low PLR had longer OS than those with high PLR, but such a difference was not found in the patients who received the SPA regimen. To the best of our knowledge, there have been limited studies on the relationship between the value of the systemic inflammation response index such as NLR and PLR and the OS of stage IV GC patients. The results of the present study indicate that the NLR–PLR score may be a significant prognostic factor in GC that can be used to stratify patients to the appropriate chemotherapy regimen. Additionally, there is no evidence for the selective superiority of the SPA regimen and the SOX regimen. In a phase II study of the SPA regimen as first-line chemotherapy for patients with metastatic or advanced GC, median PFS and OS were 5.2 months and 12.2 months, respectively (50). In a phase III Japanese study of SOX as first-line chemotherapy for advanced GC patients, the PFS and OS were 5.5 months and 14.1 months, respectively (51). To explore the therapeutic efficacy of SPA and SOX as first-line treatment for GC, we divided the patients into the SPA group and the SOX group for analyses. The results demonstrated no significant difference in prognoses for those who received these two regimens. Furthermore, there was no significant difference in the prognoses of those who received SPA and SOX when patients were stratified into high-NLR and low-NLR groups and the high-PLR and low-PLR groups (Supplementary Figure 3). These findings may suggest that there is no significant difference in efficacy between the SPA regimen and the SOX regimen when measured according to the NLR or PLR levels. However, this may also indicate that the NLR or PLR levels cannot effectively stratify patients into the appropriate chemotherapy regimen and therefore cannot be used to guide the selection of treatment regimens for advanced GC patients.

Despite profound advances in oncology, effective markers to predict the efficacy of chemotherapy are still lacking. Less-invasive tests based on a sample of body fluid (e.g., blood, urine, and saliva) that allows rapid diagnosis or treatment monitoring are urgently needed. Peripheral blood testing has the advantages of convenience, simplicity, affordability, and reproducibility. Accordingly, a comprehensive understanding of hematologic parameters may be helpful for diagnosing tumors, guiding targeted treatment, and monitoring treatment efficacy and resistance.

Although NLR and PLR have been previously reported to predict cancer prognoses, to the best of our knowledge, our study is the first to report on its prognostic role in stage IV GC and is the first to explore the association between NLR or PLR and first-line chemotherapy. However, our study also had several limitations. First, its retrospective, single-center design may have caused some potential biases. Second, patient survival varied significantly, ranging from 1 to 7 years after diagnosis, and thus there might have been biases in the results of the survival analysis. Third, there was lack of consecutive NLR or PLR counts for every patient during the first-line chemotherapy. As a dynamic marker, serial NLR or PLR measurements during treatment could potentially help to identify those patients who are not benefiting from chemotherapy at an early stage. Changes in NLR require further evaluation by clinical trials in which data can be analyzed prospectively. Fourth, the study was performed among patients with stage IV GC; therefore, the NLR–PLR score can be used only for metastatic GC. Lastly, there is no consensus on the optimal cutoff value for high and low NLR and PLR. Some studies used the median value to define the cutoff value, as in the current study; however, some studies also used the receiver operating characteristic curve. Our findings require more scientific evidence, which should be acquired through prospective multicenter trials with larger sample sizes.

## Conclusion

Our study demonstrated that the NLR, PLR, and NLR–PLR score may be a pre-treatment independent predictor of OS in stage IV GC, particularly among those receiving SOX or SPA as first-line chemotherapy. This study analysis may provide strong support for the treatment of GC with later period, and it may have important implications for selecting the optimal treatment strategy to ultimately improve or prolong OS.

## Data Availability Statement

All datasets generated for this study are included in the article/Supplementary Material.

## Ethics Statement

The collection and analysis of all samples in this study were approved by the Ethics Committee of the First Affiliated Hospital of Zhejiang University (Reference Number: 2017–802).

## Author Contributions

All authors contributed to data analysis, drafting, or revising of the article, approved the final version to be published, and agreed to be accountable for all aspects of the work. All authors have read and approved the final manuscript.

## Conflict of Interest

The authors declare that the research was conducted in the absence of any commercial or financial relationships that could be construed as a potential conflict of interest.

## Supplementary Material

Supplementary Figure 1 Kaplan–Meier survival curves for overall survival in patients stratified by PLR median for **(A)** age<60 and **(B)** age≥60 **(C)** CEA <5; **(D)** CEA≥5; **(E)** CA199 <37; **(F)** CA199≥37; **(G)** poor differentiation; **(H)** moderate-well differentiation; **(I)** BMI <18.5; **(J)** BMI 18.5–24.9 and **(K)** BMI≥25.

Supplementary Figure 2 Kaplan.Meier survival curves for overall survival according to the NLR+PLR score for **(A)** age<60; **(B)** age≥60 **(C)** CEA <5; **(D)** CEA≥5; **(E)** CA199 <37; **(F)** CA199≥37; **(G)** poor differentiation; **(H)** moderate-well differentiation; **(I)** BMI <18.5; **(J)** BMI 18.5–24.9 and **(K)** BMI≥25.

Supplementary Figure 3 Kaplan–Meier survival curves for overall survival in patients stratified by SPA or SOX regimens **(A)** the relationship of first-line chemotherapy and OS included all patients; **(B)** the relationship of first-line chemotherapy and OS via low NLR subgroup; **(C)** the relationship of first-line chemotherapy and OS via high NLR subgroup; **(D)** the relationship of first-line chemotherapy and OS via low PLR subgroup and **(E)** the relationship of first-line chemotherapy and OS via high PLR subgroup.

Supplementary Table 1 Univariate and multivariate Cox regress ion analysis for overall survival according to NLR.

Supplementary Table 2 Univariate and multivariate Cox regression analy sis for overall survival according to PLR.

## Abbreviations

NLR: Neutrophil–Lymphocyte Ratio
PLR: Platelet–Lymphocyte Ratio
GC: Gastric Cancer
OS: Overall Survival
PFS: Progression-Free Survival
CEA: Carcinoembryonic Antigen
CA199: Carbohydrate Antigen 199
BMI: Body Mass Index
SOX: Oxaliplatin plus S-1
SPA: Paclitaxel plus S-1
WHO: World Health Organization.
