# Supplementary material for: Prognostic Value of Neutrophil–Lymphocyte Ratio, Platelet–Lymphocyte Ratio, and Combined Neutrophil–Lymphocyte Ratio and Platelet–Lymphocyte Ratio in Stage IV Advanced Gastric Cancer
Source: Front Oncol. 2020 Jun 19;10:841. doi: 10.3389/fonc.2020.00841 (PMC7317009; doi:10.3389/fonc.2020.00841)

supplementary Table 1

	Univariate		Multivariate	
	HR(95%CI)	P-value	HR(95%CI)	P-value
Age(< 60 vs ≥60)	0.984(0.78-1.25)	0.892		
Sex(female vs male)	1.00(0.78-1.30)	0.973		
History of gastric cancer operation(no vs yes)	0.543(0.43-0.69)	<0.001	0.615(0.47-0.80)	<0.001
Intra-abdominal metastasis(no vs yes)	1.061(0.84-1.35)	0.624		
History of smoke(no vs yes)	1.128(0.88-1.44)	0.335		
History of alcohol(no vs yes)	1.037(0.80-1.34)	0.779		
Hypertension(no vs yes)	1.116(0.81-1.53)	0.496		
Diabetes(no vs yes)	1.352(0.86-2.11)	0.185		
CEA(< 5 vs ≥5)	1.248(0.98-1.59)	0.071	1.142(0.89-1.46)	0.293
CA199(< 37 vs ≥37)	1.070(0.84-1.37)	0.001	0.910(0.71-1.17)	0.463
NLR(low vs high)	1.915(1.51-2.43)	<0.001	1.674(1.30-2.16)	<0.001
Differentiation(poor vs moderate-well)	0.830(0.66-1.05)	0.124	0.735(0.58-0.94)	0.012

supplementary Table 2

	Univariate		Multivariate	
	HR(95%CI)	P-value	HR(95%CI)	P-value
Age(< 60 vs ≥60)	0.984(0.78-1.25)	0.892		
Sex(female vs male)	1.00(0.78-1.30)	0.973		
History of gastric cancer operation(no vs yes)	0.543(0.43-0.69)	< 0.001	0.537(0.42-0.69)	< 0.001
Intra-abdominal metastasis(no vs yes)	1.061(0.84-1.35)	0.624		
History of smoke(no vs yes)	1.128(0.88-1.44)	0.335		
History of alcohol(no vs yes)	1.037(0.80-1.34)	0.779		
Hypertension(no vs yes)	1.116(0.81-1.53)	0.496		
Diabetes(no vs yes)	1.352(0.86-2.11)	0.185		
CEA(< 5 vs ≥5)	1.248(0.98-1.59)	0.071	1.169(0.91-1.50)	0.216
CA199(< 37 vs ≥37)	1.070(0.84-1.37)	0.001	0.923(0.72-1.19)	0.538
PLR(low vs high)	1.555(1.23-1.97)	< 0.001	1.483(1.17-1.89)	0.001
Differentiation(poor vs moderate-well)	0.830(0.66-1.05)	0.124	0.741(0.58-0.94)	0.015

Supplementary Fig. 1

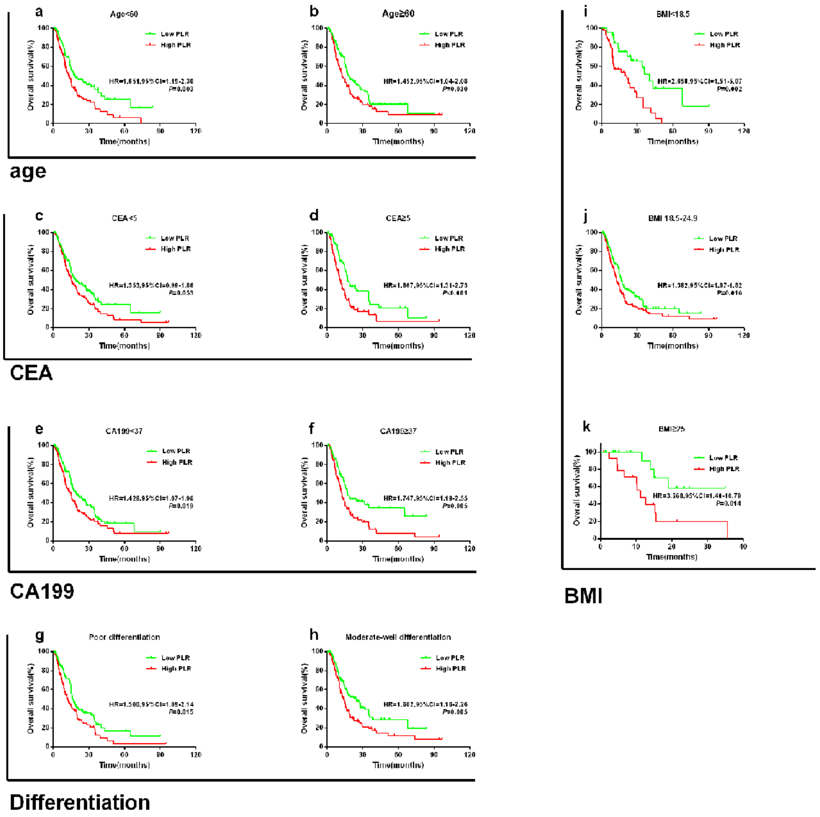

Supplementary Fig. 2

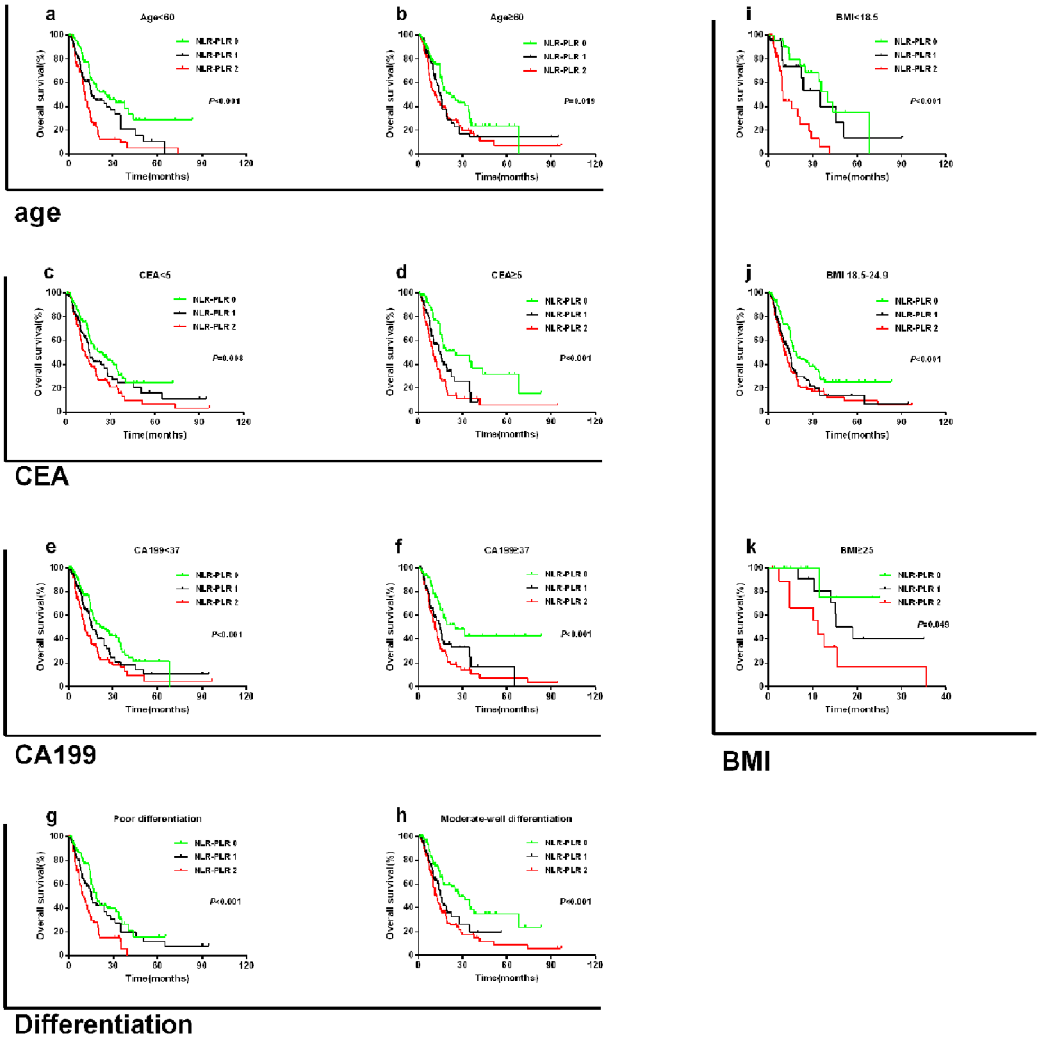

Supplementary Fig. 3

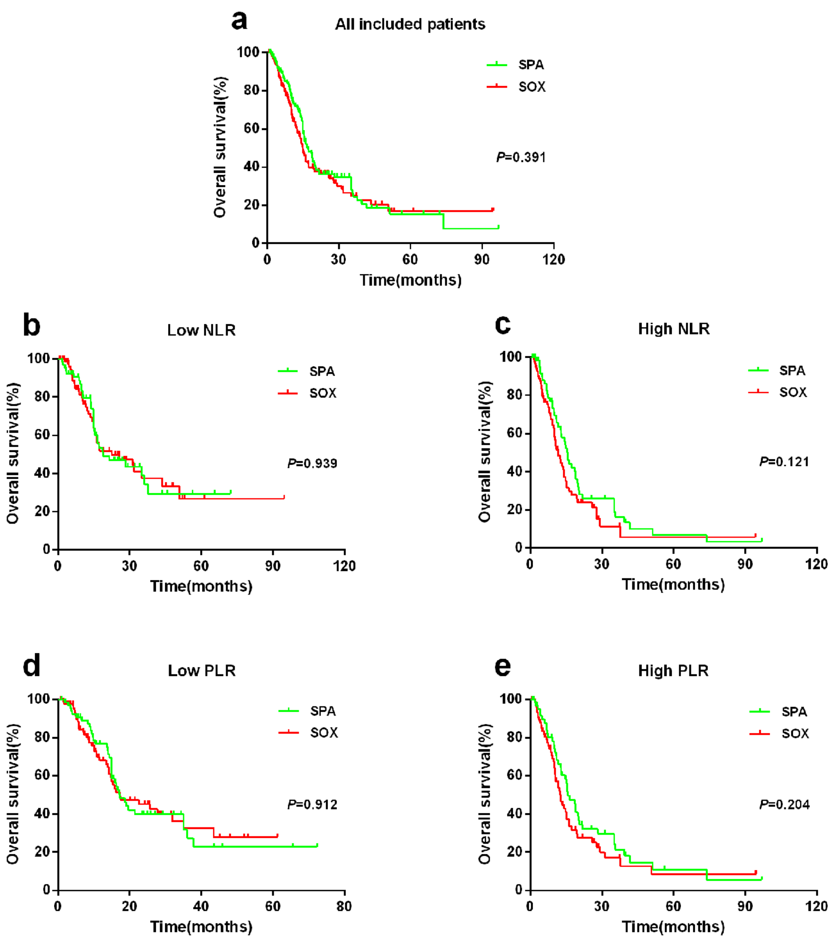

Supplement: Supplementary Figure 1 — Kaplan–Meier survival curves for overall survival in patients stratified by PLR median for (A) age<60 and (B) age≥60 (C) CEA <5; (D) CEA≥5; (E) CA199 <37; (F) CA199≥37; (G) poor differentiation; (H) moderate-well differentiation; (I) BMI <18.5; (J) BMI 18.5–24.9 and (K) BMI≥25. [file Data_Sheet_1.PDF]
